# A machine learning–coupled APSIM model pipeline for projected oil palm yield in Surat Thani, Thailand

**DOI:** 10.1371/journal.pone.0349782

**Published:** 2026-06-10

**Authors:** Napat Jantaraprasit, Parichart Promchote, Shih–Yu Simon Wang, Sugontee Daengnui, Sajad Khoshnood Motlagh, Andre Geraldo de Lima Moraes, Luthiene França, Jin–Ho Yoon, Chalermpol Phumichai, Piya Kittipadakul

**Affiliations:** 1 Department of Agronomy, Kasetsart University, Bangkok, Thailand; 2 Department of Watershed Sciences, Utah State University, Logan, Utah, United States of America; 3 Department of Plants, Soils, and Climate, S. J. Jessie E. Quinney College of Agriculture and Natural Resources, Utah State University, Logan, Utah, United States of America; 4 Department of Environment and Energy Engineering, Gwangju Institute of Science and Technology, Gwangju, South Korea; Sophia University: Jochi Daigaku, JAPAN

## Abstract

Accurate and timely forecasts of oil palm yield are essential for both short-term farm management and long-term adaptation planning, yet their reliability is often constrained by the coarse spatial resolution of climate datasets and structural biases in process-based crop models. To address these challenges, we developed an end-to-end modeling framework that integrates spatially refined climate information with a hybrid process–machine-learning approach. Our method employs Spatial Interactions Downscaling to convert reanalysis, seasonal forecasts, and CMIP6 climate projections into fine-scale datasets anchored to the CHELSA baseline. These downscaled drivers are then coupled with the Agricultural Production Systems sIMulator (APSIM) and a Random Forest (RF) model to correct residual errors and improve predictive accuracy. A case study in Surat Thani, Thailand, demonstrates the framework’s performance and utility. Downscaled climate variables showed strong agreement with CHELSA, with minimal bias and compact error distributions, especially for temperature. Stand-alone APSIM overestimated yields (RMSE = 15.51 t ha ⁻ ¹), whereas the APSIM + RF hybrid significantly improved accuracy (RMSE = 5.52 t ha ⁻ ¹ at observed sites; 2.74 t ha ⁻ ¹ when averaged across sites). Seasonal forecasts based on downscaled data achieved skill levels comparable to those driven by reanalysis, enabling reliable yield predictions up to eight months in advance. On centennial scales, CMIP6 projections suggest stable to slightly higher yields in the early 21st century, a modest mid-century decline, and late-century stabilization across scenarios. These results indicate that oil palm production in southern Thailand is relatively resilient to projected climate change. More broadly, the framework offers a transferable approach for integrating fine-scale climate information and hybrid modeling to improve crop forecasting, support climate-risk assessment, and inform adaptation strategies across agricultural systems.

## Introduction

Oil palm (*Elaeis guineensis* Jacq.) has evolved from its African origins to become a global agricultural powerhouse, underpinning food, fuel, and personal care products worldwide [1–3]. As the world’s third-largest producer [4], Thailand’s economy, particularly in the south, is heavily reliant on this crop. Surat Thani province, with over 20% of its land dedicated to oil palm, represents one of the nation’s oldest and largest cultivation areas [5]. However, the productivity of this long-lived perennial crop is increasingly threatened by climate change and weather extremes. This creates an urgent need for accurate, fine-scale yield prediction models that can help stakeholders develop adaptive strategies and ensure regional food and economic security [6,7].

Process-based models are crucial for simulating crop responses to environmental factors. The Agricultural Production Systems sIMulator (APSIM) Oil Palm module has become a versatile tool for yield prediction across diverse environments [8]. Applications span from evaluating adaptive strategies under future climates in Nigeria’s Niger Delta [9], evaluating the influence of soil and management parameters on yield and nitrogen losses [10], supporting irrigation scheduling through sensor integration in Colombia [11], simulating water demands on marginal acidic soils [12], and assessing irrigation as an adaptation option under projected climate change in Costa Rica [13]. Most applications employ the default dura × pisifera “Dami” cultivar and standard planting density of 135 palms ha ⁻ ¹, with a few variations such as the IRHO cultivar [9] and increased density [11]. Soil and climate inputs are typically derived from field observations, although some studies integrate modeled or remote-sensing data. Calibration is rarely reported, with Watson-Hernández et al. being an exception, manually adjusting soil water limits to improve model fit. Across studies, APSIM achieves acceptable to good performance, with reported yield simulations showing R² values of approximately 0.66–0.75 and RMSE in the range of 2.5–7.0 t ha ⁻ ¹ yr ⁻ ¹ [9,11,13], highlighting its effectiveness as a process-based tool for oil palm yield analysis and management planning.

Integrating APSIM with machine learning has shown substantial improvements over standalone process-based approaches. In wheat systems, APSIM–machine learning hybrids have achieved strong performance: Feng et al. (2019) captured 81% of yield variance while preventing the 1–10% overestimation typical of single-model APSIM, Shahhosseini et al. (2021) reduced relative-RMSE by 7–20%, and Bai et al. (2024) reached R² ≈ 0.93 by correcting for climate extremes. These hybrid frameworks often use crop-model internal states (e.g., soil water and nitrogen status, phenology, biomass) as inputs to machine learning to correct systematic process-model errors and improve yield prediction and generalization [14–17]. Hybrid modelling has also expanded toward knowledge-guided workflows that link agricultural system modelling with machine learning for decision support under climate stress, including spatiotemporal co-optimization of management practices to meet yield and sustainability targets [18]. These advances motivate our oil palm application, where APSIM provides process-relevant physiological and soil indicators and machine learning models residual errors under spatially independent validation and across consistent reanalysis, forecast, and scenario climate forcings.

Despite these advances, critical limitations constrain current oil palm yield prediction capabilities. First, existing gridded climate products operate at coarse resolutions (25–100 km), inadequate for farm-level decision support [19–24]. While CMIP6-driven crop modeling has been successfully implemented for rice in Thailand’s Lower Chao Phraya Basin [25], fine-scale applications for oil palm remain unreported. Second, integration of seasonal climate predictions such as CFSv2 for operational yield forecasting remains limited in oil palm systems, despite evidence that seasonal prediction may provide actionable skill for Thailand’s palm oil sector [6].

Oil palm yield forecasting has traditionally relied on statistical models linking yield to lagged weather and moisture conditions. These approaches provide useful baselines but often assume stable relationships across time and space and have limited capacity to represent non-linear responses. For example, weather-based regression models using lagged agro-meteorological variables have demonstrated practical forecast lead times in plantation settings [26], and climate-based studies have shown that large-scale modes such as ENSO can influence yield through water-stress pathways [7]. Beyond regression, probabilistic approaches such as Bayesian networks have also been applied using commercial plantation datasets to represent dependencies among environment, management, and yield components [27].

In parallel, machine learning and deep learning have been increasingly adopted to capture complex, lagged, and interacting climate–yield relationships using plantation records and agro-meteorological predictors, including workflows designed to improve explainability and operational use [28,29]. Several studies have explored a broader range of models, including tree-based methods, support vector regression, and deep neural networks when larger datasets and multi-source predictors are available [30,31]. Remote-sensing time series have also been used to estimate or predict oil palm yield by extracting vegetation and water-stress signals that precede harvest, and reviews highlight the expanding use of remote sensing for plantation monitoring and yield-related applications [32,33].

Despite the growing use of ML/DL, published oil palm yield studies have generally remained either process-based simulation studies (e.g., APSIM applications) or purely data-driven ML/DL studies using climate, agronomic, or remote-sensing predictors [8,9,29–31,34]. As a result, there is limited evidence on process-guided hybrid oil palm yield frameworks that leverage crop-model internal states to improve out-of-location performance while retaining physiological interpretability, and that are applied consistently across historical reanalysis, seasonal forecasts, and CMIP6 scenario projections. This study addresses these gaps by coupling APSIM-derived physiological and soil indicators with machine learning, using spatially independent validation to assess transferability, and applying the unified framework to reanalysis-, forecast-, and scenario-based climate inputs for yield forecasting and projection at fine spatial scales.

Scientific contributions of this study are as follows. [1] We propose an integrated modeling workflow that links APSIM simulations with a RF model to predict annual oil palm yield, leveraging APSIM to represent crop–climate processes and machine learning to correct residual errors and capture non-linear relationships. [2] We provide a systematic comparison of APSIM, RF, linear regression, and APSIM + RF using the same evaluation framework. [3] We apply the validated framework to fine-scale yield forecasting and to future yield projection under CMIP6 SSP1–2.6, SSP2–4.5, and SSP5–8.5, translating climate uncertainty into yield-relevant outcomes. These advances are important because they improve the reliability of oil palm yield forecasts and projections, supporting decision-making for management, investment, and adaptation under increasing climate variability and change.

## Materials and methods

### Oil palm yield and cultivation data

Annual oil palm yield records, cultural practices, and geographical coordinates for 20 farms in Surat Thani Province, Thailand (2015–2023) were provided by the Tapi–Ipun Sustainable and Lumnam Kadae Pattana Oil Palm Community Enterprise Groups under the Roundtable on Sustainable Palm Oil (RSPO). The dataset represents plantations averaging 19 years of age. Oil palm cultivation areas were identified from the CROPGRIDS database [35], with non-arable land (e.g., sea and protected forests) masked to accurately represent cultivated zones. These coordinates were spatially matched to climate and soil datasets to ensure that modeling conditions reflected real on-farm environments (S1 Table).

### Climate data preparation

The APSIM-Oil Palm module [8] requires daily inputs of precipitation (PR), maximum temperature (TASMAX), minimum temperature (TASMIN), and downward surface solar radiation (RSDS). Fine-scale reanalysis data (0.03° resolution) from CHELSA-W5E5 v1.0 [36,37] were used as the historical baseline, covering the period 1979–2016 (S1 Table). Preliminary analysis showed no significant differences between CHELSA and local weather-station records (Thai Meteorological Department: TMD), so CHELSA was adopted as the observed dataset. To extend the temporal range, hourly climate data from ERA5 [20] at 0.25° resolution were incorporated, spanning 1940 to the present. In addition, seasonal forecasts were obtained from the CFSv2 system [22], which provides 1° resolution forecasts initialized from April 1–5 with four forecasts per day and 20 ensemble members per year, covering the period 2012–2025. To assess future yield projections, we used five CMIP6 GCMs from the NEX-GDDP-CMIP6 archive at 0.25° resolution [25]: ACCESS-ESM1–5, CNRM-CM6–1, EC-Earth3-Veg, MPI-ESM1–2-LR, and MRI-ESM2–0 (S1 Table).

### Downscaling and bias correction

All coarse-resolution climate datasets (ERA5, CFSv2, and CMIP6) were downscaled to the fine spatial scale of CHELSA using the Spatial Interactions Downscaling (SPID) approach [38]. In this process, the CHELSA dataset was first upscaled and spatially aligned with each coarser dataset (e.g., Up CHEL–ERA and Up CHEL–CFSv2) to ensure consistent grid structures. ERA5 data were then bias-corrected using quantile mapping against the Up CHEL–ERA reference for the 1986–2016 period, with the correction extended through early 2025. Likewise, CFSv2 forecasts were resampled from hourly to daily resolution and bias-corrected against the Up CHEL–CFSv2 reference for 2011–2016, with the corrected dataset similarly extended to 2025.

A Python implementation of SPID was used: for each CHELSA grid cell (predictant), a RF model trained on 70% of data used ten upscaled predictors (nine surrounding cells plus one overlay cell). When root mean square error (RMSE) (Equation (1)) bias was < 5%, models were retrained on the full dataset and applied to generate final downscaled fields. Validation covered 1980–2010 for ERA5 and CMIP6, and 2012–2016 for CFSv2, reflecting their respective data availability.


$$\begin{array}{c} \text{RMSE}=\sqrt{\frac{\text{1}}{\text{n}}\sum_{\text{i}=\text{1}}^{\text{n}}{\left({\text{y}}_{\text{i}}-\;{\hat{\text{y}}}_{\text{i}}\right)}^{\text{2}}\;} \end{array}$$ (1)

### Soil and land surface data

1 Soil profiles were obtained from the Global Soil Dataset (GSDE) [39]. Saturated hydraulic conductivity (Ks) was estimated using a pedotransfer function [40], and saturated water content (SAT) (Equation (2)) [41]. CROPGRIDS was regridded from 0.05° to 0.03° using nearest-neighbor mapping [42], and CHELSA grids were masked to delineate oil palm areas. These zones served as extraction points for soil profiles and downscaled climate time series, forming the spatial domain for APSIM simulations.

$$\begin{array}{c} \text{SAT}=(\text{1}-\frac{\text{BD}}{\text{2}.\text{65}})-\text{0}.\text{03} \end{array}$$ (2)

Equation after Dalgliesh and Foale (1998): BD = bulk density

### APSIM simulation setup

Planting density was set to 143 trees ha ⁻ ¹ based on equilateral triangular spacing (9.5 m). Sowing was scheduled for May 1 (onset of the rainy season) following regional recommendations [43,44]. We selected Univanich’s oil palm cultivar for Surat Thani province [**45]**, as it was the predominant type represented in the RSPO dataset. Several APSIM cultivar parameters were adjusted using data from the Department of Agronomy’s oil palm experimental field at Kasetsart University, Saraburi, Thailand. Adjusted traits included maximum potential bunch weight (g), maximum leaf area of a single frond (m²), number of fronds retained at harvest, fraction of assimilates allocated to stem versus fronds, maximum leaf area per unit dry mass (m² g ⁻ ¹), and maximum nitrogen concentration in fronds (%) (S3 Table). For farm-level simulations, sowing dates matched RSPO observations. For province-scale simulations, sowing was fixed to May 1, 2003, corresponding to the average stand age [46]. Fertilization followed Department of Agriculture guidelines [44] (S4 Table). Surface organic matter inputs represented a 27.5-year-old plantation, with frond biomass of 14,100 kg ha ⁻ ¹ (C:N = 41.38) and trunk + root biomass of 53,700 kg ha ⁻ ¹ (C:N = 176.1) [47].

### Reanalysis and seasonal forecast yields

**Fig 1:**
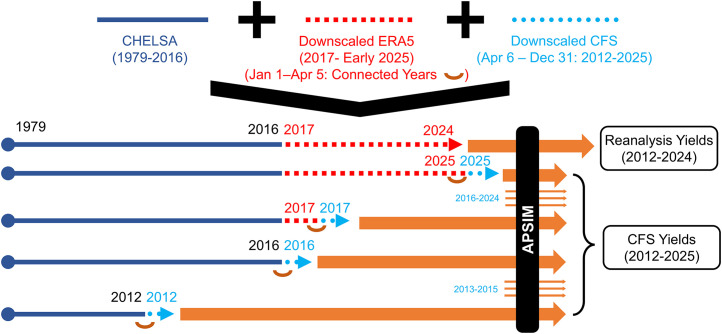
Diagram shows climate dataset concatenations before simulation in APSIM.

We created two distinct datasets. For Reanalysis yields, we concatenated CHELSA (2003–2016) with downscaled ERA5 (2017–Early 2025). For nine-month seasonal forecast yields (CFSv2), we built a composite dataset by combining CHELSA (2003–2016) with downscaled ERA5 (2017–Early 2025). The last year of downscaled ERA5 was limited to Jan 1–Apr 5 and connected with downscaled CFSv2 from Apr 6–Dec 31. This combination was repeated until the period 2012–2025 was fully covered (Fig 1). Yield simulations for the 2025 forecast year were also included in this study.

Because the final climate drivers are formed by concatenating multiple products, we evaluated potential discontinuities at the CHELSA–ERA5 and ERA5–CFSv2 junctions. Bias correction is anchored to CHELSA via quantile mapping during overlap periods, which aligns the distributions before concatenation. We additionally examined cultivated-area averages across the transition periods as a continuity diagnostic (S3 Fig and S2 Table), and we found no evidence of abrupt step changes in the concatenated time series.

### Projected yield

The APSIM Oil Palm module simulates growth up to 23 years, and oil palm exhibits distinct juvenile and mature yield phases. Juvenile palms typically show rapid yield increases until maturity, while mature palms (commonly reported around 7–18 years after planting) maintain relatively stable yields before gradually declining toward replanting [48,49]. To align with both APSIM behavior and the literature, we define the mature-yield period as 10–18 years after planting (a 9-year window). To reduce sensitivity to establishment and senescence transients, all historical and future summaries are computed using this same mature-window definition. We acknowledge that shifting the maturity window by a few years can change absolute mean yields; however, our climate-change assessment focuses on differences relative to the historical baseline using the same window definition, which reduces sensitivity in relative comparisons. We discuss this uncertainty and recommend age-structured or multi-window sensitivity analyses in future work.

Yield simulations were performed using CMIP6 climate datasets, and mature yields were extracted for four time intervals, consistent with Vilavan et al. (2024), across all GCMs:

1. Historical (2014–2022)
2. Early century (2032–2040)
3. Mid century (2062–2070)
4. Late century (2092–2100)

**Fig 2:**
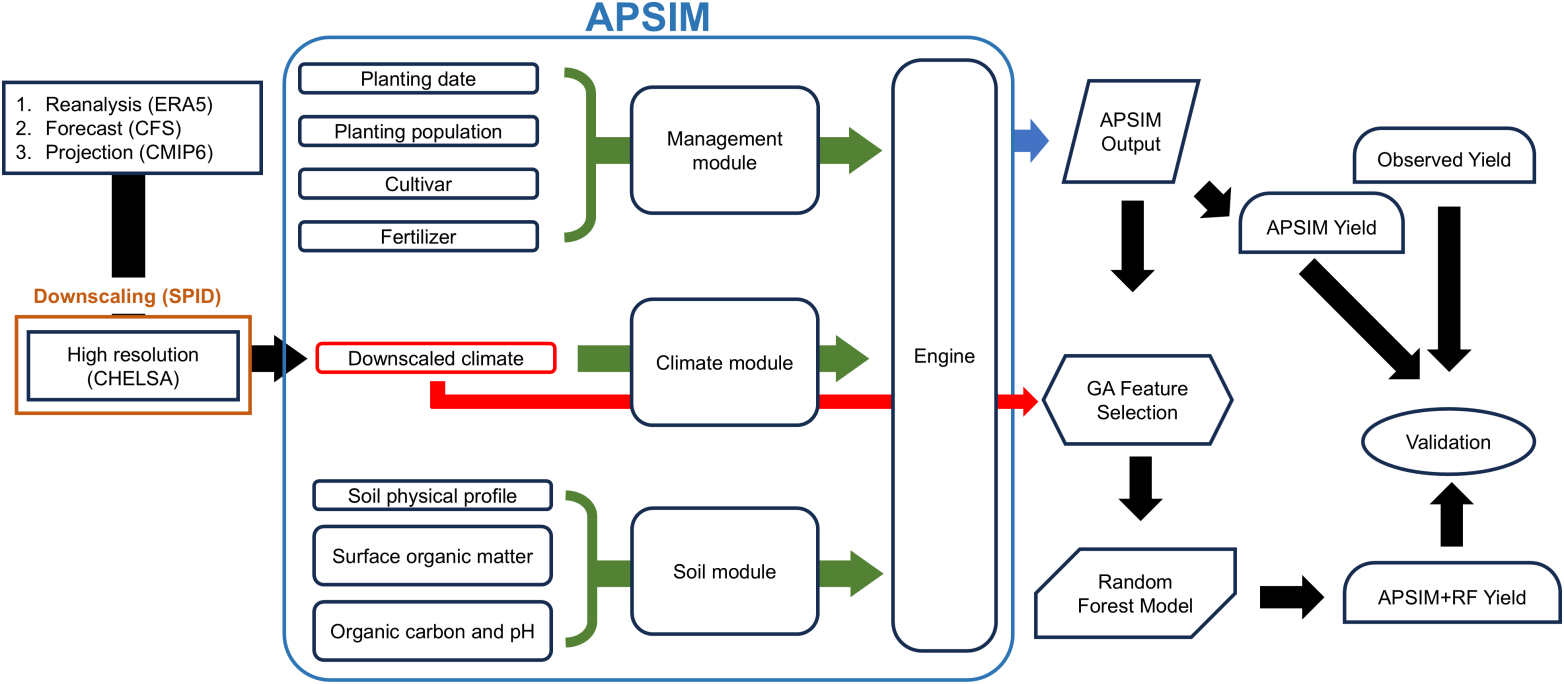
Systematic research workflow from initial dataset preparation through final oil palm yield simulation adapted from Watson– Hernández et al. (2023).

The results from individual GCMs were combined into an ensemble for each scenario. To assess differences, we applied the Mann–Whitney U test to compare historical yields with future projections (9 mature–year samples per pair, spatially) [50]. Additionally, a Kruskal–Wallis test was used to evaluate median differences across the four intervals [51]. The research methodology schematic diagram is presented in Fig 2.

### APSIM-machine learning coupled yield simulation

Crop biomass, crop physiological traits, simulated yield, net primary productivity, and soil water–nutrient profiles were extracted from APSIM outputs, and four climate variables (TASMAX, TASMIN, PR, and RSDS) were integrated. Plant–available soil water in each layer and all climate predictors was lag–adjusted by 1–3 years (S5 Table), after which a Genetic Algorithm (GA) was applied to refine the predictors and reduce dimensionality. GA, inspired by natural selection, evolves candidate solutions through selection, crossover, and mutation until near–optimal subsets are identified [52]. In this study, GA was configured with a population size of 50, a crossover rate of 0.8, and a mutation rate of 0.1 [16,53].

The simulated yield data from observed sites were divided into 80% training and 20% testing, with the test set reserved for final validation. On the training data, we implemented a Leave–One–Location–Out (LOLO) strategy with a 10–fold cross–validation inner loop [54], where all observations from one site were held out as the validation fold. This spatial validation design reduces spatial leakage and provides a more realistic estimate of model transferability across locations where yield–climate relationships may differ. RF was selected over more flexible architectures because each LOLO training fold contains approximately 16 annual site-year observations, a regime where deep learning models face greater overfitting risk than RF under spatially independent evaluation. GA was run across all LOLO folds, and the feature subset yielding the lowest RMSE was retained. A RF model was then trained using this feature set, with hyperparameter tuning performed via GridSearchCV [31]. The configuration achieving the minimum cross–validated RMSE was selected for subsequent testing. We used RMSE as the primary model-selection criterion because it is expressed in the same units as yield (t ha ⁻ ¹ yr ⁻ ¹) and directly measures the typical magnitude of prediction error, which is the most interpretable quantity for forecasting applications and operational decision-making. To complement RMSE, we additionally report relative RMSE (RRMSE) (Equation (3)) to enable scale-normalized comparisons across sites/years with different mean yields, and mean bias error (MBE) to quantify systematic over- or under-estimation that RMSE alone cannot reveal. The finalized RF model was then applied to independent APSIM simulations forced by Reanalysis, CFSv2, and CMIP6 climate datasets, and performance was evaluated using RMSE, RRMSE, MBE (Equation (4)), and R² (Equation (5)). For comparison, simulations based on Reanalysis data were additionally bias-corrected using both delta-mean and quantile-mapping approaches [55].


$$\begin{array}{c} \text{RRMSE}=\;\frac{\text{RMSE}}{\text{Mean observed}}\;\times \text{100} \end{array}$$ (3)

$$\begin{array}{c} \text{MBE}=\;\frac{\text{1}}{\text{n}}\sum_{\text{i}=\text{1}}^{\text{n}}\left({\text{y}}_{\text{i}}-\;{\hat{\text{y}}}_{\text{i}}\right) \end{array}$$ (4)

$$\begin{array}{c} {\text{R}}^{\text{2}}=\;\text{1}-\frac{\sum_{\text{i}=\text{1}}^{\text{n}}{\left({\text{y}}_{\text{i}}-\;{\hat{\text{y}}}_{\text{i}}\right)}^{\text{2}}}{\sum_{\text{i}=\text{1}}^{\text{n}}{\left({\text{y}}_{\text{i}}-\;\overset{―}{y}\right)}^{\text{2}}}\;\times \text{100} \end{array}$$ (5)

Because observed yields are annual time series and oil palm exhibits carry-over effects, observations within a site may be serially correlated. Our validation design is therefore intentionally spatially grouped. The LOLO procedure holds out all years from one site at a time, which prevents within-site temporal leakage into validation folds and yields an out-of-location performance estimate. Temporal dependence within training sites can reduce the effective sample size, so we interpret cross-validated RMSE values cautiously and primarily as an indicator of spatial transferability. A lag-1 autocorrelation correction applied to training residuals confirmed that the effective sample size remained above 60% of the nominal count; autocorrelation reduces but does not invalidate the out-of-location RMSE estimates.

To interpret the model, we employed Shapley Additive Explanations (SHAP) to quantify the contribution of individual features [56]. SHAP, a game theory–based framework, explains machine learning predictions by assigning each feature a value that represents its effect on the output. Specifically, a SHAP value indicates how much a given feature shifts an individual prediction away from the overall average prediction, thereby showing its influence on the predicted yield [56]. The final model was applied for spatial yield simulations under downscaled Reanalysis, CFSv2, and CMIP6 climate datasets.

## Results

### Performance of the downscaled climate datasets

**Fig 3:**
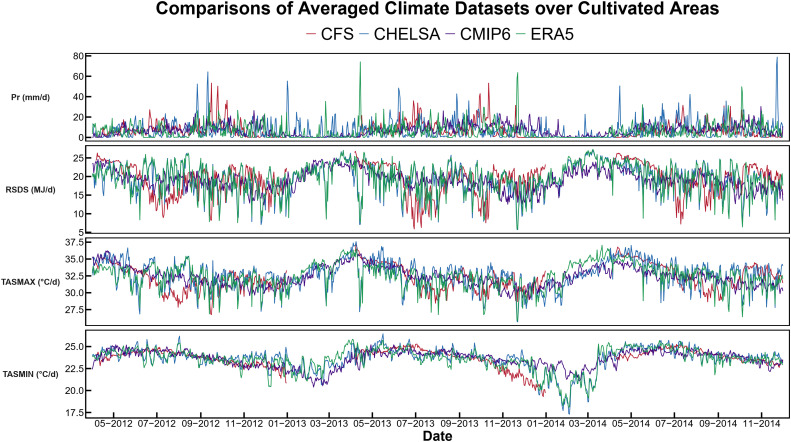
Line chart comparison of spatially averaged climate datasets over cultivated areas in Surat Thani used for APSIM simulations (2012–2014). Note: CFSv2 datasets are available from April–December.

**Fig 4:**
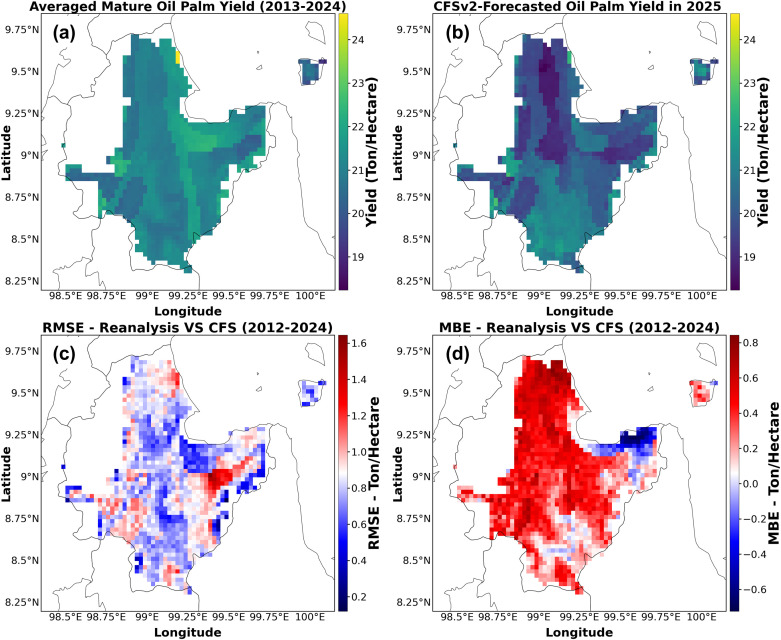
Spatial distribution of mature oil palm yield: (a) averaged yield (2013–2024),(b) CFSv2–forecasted yield for 2025, (c) RMSE between Reanalysis and CFSv2 (2012–2024), and (d) mean bias error (MBE) between Reanalysis and CFSv2 (2012–2024). Provincial boundaries from SimpleMaps.com, licensed under CC BY 4.0.

All downscaled climate products show sufficient spatial accuracy and temporal consistency to serve as reliable inputs for APSIM simulations. ERA5 exhibits the closest agreement with CHELSA, with small, spatially uniform errors across all variables. CFSv2 shows slightly larger, more structured biases in TASMAX, TASMIN, and RSDS, while PR variability is highest overall (S1 Fig), though errors remain mostly confined to mountainous regions outside cultivated areas (Fig 4) (Tang et al. 2024). The downscaled CMIP6 ensemble captures TASMAX, TASMIN, and RSDS patterns well, with PR errors more dispersed but still within acceptable ranges (S2 Fig). Temporal comparisons over cultivated areas in Surat Thani (Fig 3) reinforce these results. ERA5 closely follows CHELSA in both magnitude and timing, while CFSv2 and CMIP6 successfully capture key seasonal signals. CFSv2 tends to underestimate PR and smooth out extreme events but still reproduces the seasonal progression of rainfall and temperature. CMIP6 generally aligns with observed dynamics but slightly underrepresents features like the mid-2013 TASMIN dip.

CFSv2 shows strong predictive skill in the early and mid-season, with low TASMAX, TASMIN, and RSDS errors and minimal bias (Table 1). PR is typically underestimated during the monsoon onset, but seasonal totals remain close to observations. Forecast reliability decreases slightly toward the late monsoon, especially for TASMIN and PR, while TASMAX and RSDS stay relatively stable. Overall, spatial and temporal evaluations confirm that ERA5, CFSv2, and CMIP6 effectively reproduce the key climate signals driving oil palm growth (Fig 3, S1, and S2 Fig; Table 1), making them reliable climate drivers for APSIM simulations and supporting robust yield modelling.

**Table 1:** Monthly and seasonal performance metrics of CFSv2 relative to CHELSA over the April–December forecast window. TASMAX, TASMIN, and RSDS metrics are calculated from daily data, while precipitation (PR) metrics are based on monthly totals.

	RMSE				MBE			
Target	TASMAX (°C)	TASMIN (°C)	PR (mm)	RSDS (MJ m ⁻ ²)	TASMAX (°C)	TASMIN (°C)	PR (mm)	RSDS (MJ m ⁻ ²)
**Apr**	1.91	0.92	101.71	4.40	0.87	−0.35	−86.07	2.79
**May**	2.11	0.88	117.88	4.74	0.52	−0.62	−98.24	3.25
**Jun**	2.26	0.7	93.85	5.63	0.68	0.18	49.55	−0.68
**Jul**	2.11	0.73	119.13	6.22	−0.87	0.32	37.34	−3.71
**Aug**	2.42	0.74	104.97	4.30	−1.10	−0.20	−83.92	−0.87
**Sep**	2.03	0.6	66.79	4.96	−0.20	−0.21	31.53	0.72
**Oct**	2.12	0.62	162.75	4.80	0.32	−0.39	38.24	1.66
**Nov**	2.09	1.39	187.49	5.14	0.33	−1.13	−162.87	2.69
**Dec**	2.49	2.27	126.08	5.02	0.28	−1.74	−117.79	2.05
**Apr-Jun**	2.11	0.83	206.22	4.98	0.68	−0.26	−134.76	1.75
**Jul-Sep**	2.2	0.69	139.38	5.22	−0.73	−0.03	−15.04	−1.31
**Oct-Dec**	2.24	1.58	342.63	4.99	0.31	−1.09	−242.43	2.13

### Feature importance and performance of the APSIM + RF hybrid model

The genetic algorithm (GA) selected 19 predictors that minimized cross–validated RMSE for the hybrid APSIM + RF framework. Subsequent SHAP analysis highlighted the combined importance of climatic, soil, and process–based variables in driving oil palm yield predictions (S6 Table). Solar radiation was the single most influential factor, with the previous year’s radiation accounting for the largest share of model variation (12.5%). Temperature also contributed strongly, particularly maximum temperature at multi–year lags, underscoring the role of longer–term warming patterns in shaping yield outcomes. Among APSIM–derived indicators, oil palm bunch net primary production and simulated yields ranked within the top five features, demonstrating the value of process–based outputs in enhancing model performance. Soil nitrogen availability was another consistent driver, with nitrate concentrations at multiple depths, total nitrogen, and organic nitrogen all exerting strong predictive influence (S6 Table).

Reanalysis-driven APSIM consistently overestimated yields. Bias correction using the delta-mean and quantile-mapping approaches reduced errors but left substantial residuals, with RMSE remaining > 11.5 t ha ⁻ ¹ and R² remaining negative for all bias-corrected APSIM variants (S7 Table). Standalone statistical and RF models also showed limited explanatory skill reducing RMSE to around 6.5 t ha ⁻ ¹, yet R² remained negative (S7 Table). By contrast, the APSIM + RF hybrid performed consistently well across climate inputs, with the reanalysis-driven hybrid ranking best overall and CFSv2 and CMIP6 scenarios showing comparable skill (S7 Table). Overall, improvements in R² and reductions in error were observed only when APSIM was coupled with machine learning, not for APSIM-only or machine-learning-only models (S7 Table). APSIM + RF is the only configuration to achieve positive R² under spatially independent validation (0.17 across sites; 0.35 site-averaged). All other configurations return negative R², confirming that neither process-based nor data-driven modeling alone explains yield variance beyond the observed mean.

### Spatial yield distribution and skill of seasonal forecasts

Averaged Reanalysis-driven yields for mature oil palm during 2013–2024 ranged from 19 to 24 t ha ⁻ ¹ yr ⁻ ¹, with relatively uniform spatial patterns and slightly higher productivity in the eastern zone (Fig 4a). The eight-month lead CFSv2 forecast for 2025 reproduced these large-scale gradients, projecting a spatial mean of 20.18 t ha ⁻ ¹ yr ⁻ ¹, or −0.96 t ha ⁻ ¹ relative to the baseline (Fig 4b). Despite the long lead time, the forecast captured key spatial yield patterns, indicating that climatic signals relevant to oil-palm productivity remain predictable months in advance. Validation against the Reanalysis baseline (2012–2024) showed low RMSE values (< 0.8 t ha ⁻ ¹) across most cultivated areas (Fig 4c), demonstrating skill in reproducing spatial variability and interannual fluctuations. The MBE map revealed a consistent positive bias of > 0.2 t ha ⁻ ¹, with only small eastern zones showing slight underestimation (Fig 4d).

Performance metrics further demonstrate that the APSIM + RF hybrid can translate long-lead CFSv2 forecasts into reliable annual yield predictions. Across four validation sites, the CFSv2-driven model achieved an RMSE of 5.71 t ha ⁻ ¹ and an RRMSE of 27.12% (S7 Table), only slightly higher than the reanalysis-driven benchmark (5.52 t ha ⁻ ¹ and 26.21%) (S7 Table). Site-averaged results were similarly close (3.00 t ha ⁻ ¹ and 14.23% vs. 2.74 t ha ⁻ ¹ and 13.01%) (S7 Table), indicating that even with eight-month lead inputs, the hybrid model captures most interannual yield variability.

Although APSIM simulates yield dynamics continuously, this study focuses on aggregated annual outcomes, meaning that errors in different parts of the season accumulate into the final yield estimates. In this context, forecast skill early in the season (April–June) (Table 1) when temperature and radiation are most accurately predicted plays a disproportionate role in shaping the overall yield signal. Mid-season conditions (July–September) (Table 1) remain well represented and contribute to stable predictive performance, while late-season uncertainties, particularly in TASMIN and PR (October–December) (Table 1), introduce some variability into annual results. Despite these limitations, the forecasts retain sufficient accuracy for decision-making, demonstrating that seasonal climate information, even at long lead times, can underpin robust annual yield projections when integrated into a process-guided hybrid framework.

### Projected oil palm yields under CMIP6

**Fig 5:**
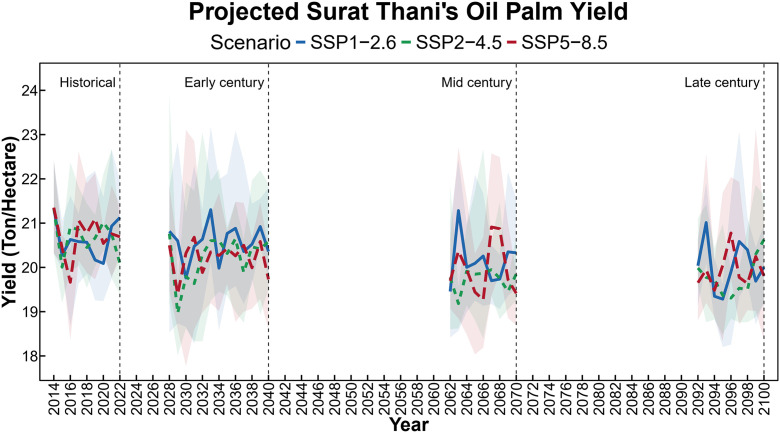
Projected spatially averaged ensemble yields under CMIP6 scenarios (SSP1–2.6, SSP2–4.5, SSP5–8.5), 2014–2100. Solid lines are annual ensemble means after combining GCMs within each scenario and spatially averaging.

**Fig 6:**
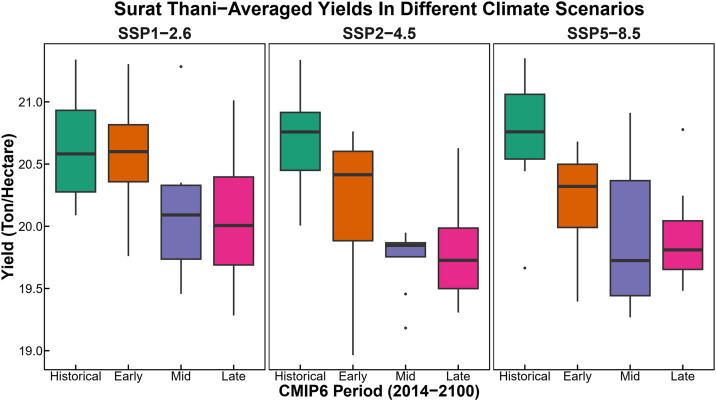
Boxplots of spatially averaged ensemble yield by period within each scenario.

Across the CMIP6 ensemble, Surat Thani’s ensemble–mean provincial yields are stable to slightly higher in the Early century (2032–2040), dip modestly in Mid century (2062–2070), and show a small recovery in Late century (2092–2100) (Figs 5 and 6). Ensemble means cluster near 20–21 t ha ⁻ ¹ yr ⁻ ¹ in the Historical period (2014–2022), increase slightly in Early century, then soften in Mid century. By Late century, SSP1–2.6 tends to recover toward Early century levels, SSP2–4.5 remains close to its Mid century median, and SSP5–8.5 remains comparatively lower (Figs 5 and 6).

Nonparametric tests corroborate a temporal signal within scenarios and weak separation among scenarios within any single period. Scenario–wise Kruskal–Wallis tests across periods are significant for all three SSPs (p < 0.05; S8 Table), consistent with the Mid century dip and Late century stabilization or recovery. Period–wise comparisons across scenarios are not significant for Historical, Mid, or Late century (p > 0.05; S8 Table), and Early century is borderline (p = 0.0502; S8 Table). Pooling all periods also yields no significant scenario separation (p = 0.161; S8 Table). Overall, timing within the century, rather than scenario choice, explains most of the projected differences at provincial scale, with ensemble medians remaining near the historical band and scenario contrasts at fixed time slices largely contained within interannual variability and model spread.

This weak provincial-scale separation is itself informative. Rather than indicating a lack of model sensitivity, it suggests that oil palm production in Surat Thani is comparatively resilient to the range of climatic changes represented by the CMIP6 scenarios. In this sense, the modeling framework serves as a robustness filter for climate risk, showing that projected climatic forcing does not translate into large provincial-scale yield disruption under most scenarios.

**Fig 7:**
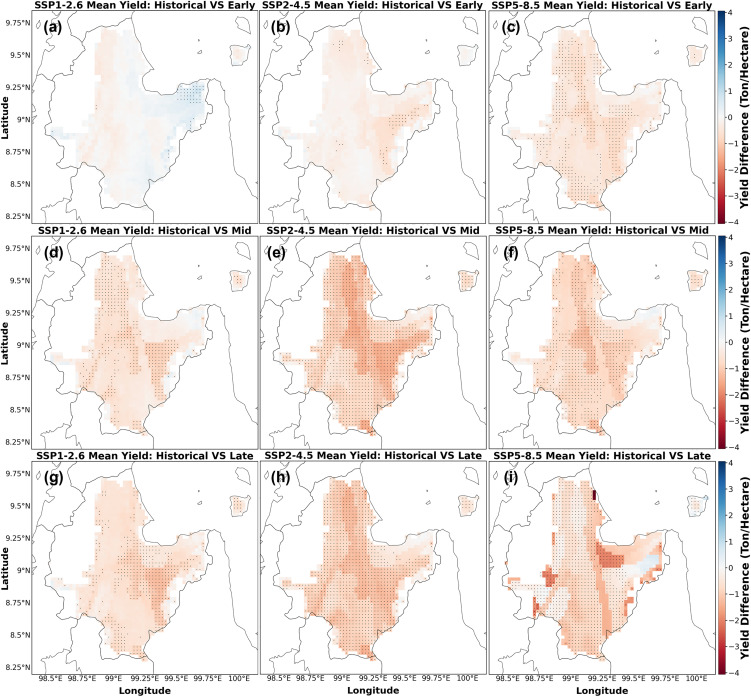
Projected yield changes under SSP1–2.6, SSP2–4.5, and SSP5–8.5 scenarios. Shading represents the magnitude of change relative to the historical baseline (2014–2022), with black dots indicating statistically significant differences (Mann–Whitney U test, p < 0.05). Provincial boundaries were reproduced from Simplemaps under a CC BY 4.0 license.

Spatially, mean yield changes are generally small, with most areas showing near-neutral to slightly negative changes relative to the historical baseline (Fig 7). Early-century changes are limited in magnitude, with small localized gains in the east under SSP1–2.6 and little evidence of widespread decline. Southern areas remain comparatively stable across scenarios and periods. By Mid century, negative changes become more spatially extensive under SSP2–4.5 and SSP5–8.5, with the central north-south belt showing the most consistent susceptibility. Late-century patterns are broadly similar, although SSP5–8.5 displays localized hotspots of stronger decline.

Although many cultivated cells show statistically significant differences in the grid-cell analysis (Mann-Whitney U test, p < 0.05), these p-values should be interpreted cautiously because large spatial sample sizes can produce statistical significance even when effect sizes are small. We therefore interpret the spatial results primarily in terms of magnitude, consistency, and spatial pattern, rather than statistical significance alone. Taken together, the CMIP6 projections indicate limited provincial-scale yield sensitivity but identifiable local hotspots of change, supporting an overall picture of relative climatic resilience in Surat Thani oil palm systems.

## Discussion

### Uncertainty in climate forcings and model robustness

The downward surface solar radiation (RSDS) values derived from ERA5 reflect the structural uncertainties typical of reanalysis in tropical regions. Because RSDS is a model-derived variable rather than an assimilated observation, it is highly sensitive to the parameterization of sub-grid scale convective clouds. In southern Thailand, the high variability of cloud cover often leads to the ‘borderline’ positive biases noted in this study, a phenomenon well-documented in previous evaluations of ERA5 performance in the tropics [57,58].

Similarly, the high RMSE and negative bias in precipitation forecasts are consistent with the known limitations of seasonal prediction systems like CFSv2 in Southeast Asia. The complex topography of the Malay Peninsula and the intermittency of the Asian Summer Monsoon present significant challenges for coarse-resolution models in capturing the exact timing and magnitude of convective events [22,59].

However, a key strength of the end-to-end pipeline presented here is its resilience to these forcing uncertainties. By utilizing a Genetic Algorithm for feature selection and an ensemble-based RF model, the framework identifies the most stable physical signals (e.g., lagged radiation and nitrogen profiles) and corrects for systematic biases in the APSIM output. Consequently, the model provides operationally viable yield forecasts even when forced with imperfect climate data, reflecting the real-world conditions of seasonal climate services.

### Performance of APSIM and machine learning models

Our results are consistent in both magnitude and error behavior, while differing in scope and validation design. Provincial means near 21 t ha ⁻ ¹ yr ⁻ ¹ align with the simulated means for Papua New Guinea sites, which cluster around 21–22 t ha ⁻ ¹ yr ⁻ ¹ and match fertilizer-trial ranges [10]. APSIM alone tends to overpredict yield with a positive bias of about 20 percent for a Nigerian estate where RMSE = 3.99 t ha ⁻ ¹ [9], while our uncorrected APSIM showed larger errors that were reduced but not eliminated by bias correction (S7 Table). After coupling APSIM with RF, our four-site RMSE was 5.52 t ha ⁻ ¹, which is within the range of calibrated APSIM in Costa Rica where RMSE spanned about 5.4–7.0 t ha ⁻ ¹ [13].

Our analysis demonstrates, for the first time, the effectiveness of coupling APSIM with machine learning for oil palm yield prediction. While APSIM alone produced high errors (RMSE = 15.51 t ha ⁻ ¹), the APSIM + RF hybrid substantially reduced error, with an average RMSE of 2.74 t ha ⁻ ¹ across the test set and 5.52 t ha ⁻ ¹ when evaluated across four independent test partitions. These improvements are consistent with evidence from other crops where APSIM–ML integrations have markedly outperformed stand-alone APSIM. Nevertheless, our RMSE remains higher than those typically reported for cereals, where hybrids have achieved RMSE values as low as 0.4–0.7 t ha ⁻ ¹ and R² up to 0.93–0.99 [16,17,60]. This gap is not unexpected, as oil palm is a perennial tree crop with strong carry-over effects, where yield reflects not only current-season climate but also cumulative environmental and management influences on fruit bunch initiation, development, and abortion. Such lagged responses make yield prediction inherently more complex than for annual crops, where yield is largely determined within a single growing cycle [6,61,62].

Feature selection and SHAP highlight radiation as the dominant driver of yield variability at provincial scale, with previous-year RSDS contributing the largest share of model variation (S6 Table). Multi-year TASMAX lags rank highly, which is consistent with cumulative heat effects on floral initiation, source capacity, and reproductive allocation [61,63]. APSIM-derived bunch NPP and simulated yields appear among the top predictors, indicating that process-based signals provide informative constraints for the RF learner. Soil nitrogen variables at multiple depths and plant available water at 45–175 cm underline the role of subsoil nutrient and water stores in buffering dry spells and supporting bunch formation [11,13]. Together, these patterns support a physiology-consistent narrative in which radiation and accumulated heat shape potential yield, while subsoil resources modulate realized yield.

### Oil palm yield performances under CFSv2 and CMIP6

Eight–month lead forecasts from the CFSv2 dataset often exhibit smaller yield discrepancies compared to those derived from the Reanalysis data. In particular, the spatial distribution of MBE and RMSE (Fig 4C and 4D) highlights that most inland areas maintain relatively low errors, suggesting that both Reanalysis and CFSv2‐based simulations capture the dominant climate patterns affecting oil palm yield. Despite climate-side biases in CFSv2 (Fig 3 and Table 1), the yield forecasts reproduced spatial gradients with modest RMSE and a small positive bias. The overall yield forecast accuracy across most of Surat Thani remains sufficiently high to support practical applications in agricultural planning. The generally small MBE, less than 0.08 t ha ⁻ ¹on average (Fig 4D), indicates that CFSv2‐based predictions do not systematically over- or underestimate yields over large areas.

Projected oil palm yields are relatively stable across time and scenarios (Figs 5–7). These results contrast with Okoro et al. (2017) in the Niger Delta, where projected mean yields increased by 30–45% after the early century. The divergence is partly methodological: Okoro incorporated dynamic adaptations such as earlier planting, irrigation, and additional fertilizer, which elevated yields beyond the baseline, whereas our projections hold management constant to isolate the direct climate signal. Okoro noted that omitting CO₂ fertilization effects and relying on bias-corrected projected inputs constrained the magnitude of negative climate impacts. Together, the studies imply that while climate alone tends to suppress or stabilize yields in tropical oil palm systems, proactive management interventions can substantially alter trajectories, underscoring the importance of adaptation in offsetting mid- to late-century climate stress.

Across cultivated areas, CMIP6 projections indicate steady warming of TASMAX and TASMIN, PR that remains near typical plantation requirements, and RSDS that stays above photosynthetically adequate levels, which together help explain why modeled yields are relatively stable. Oil palm functions well when mean temperatures are about 24–28 °C, daily maxima are about 30–32 °C, and radiation is at least 16–17 MJ m ⁻ ² day ⁻ ¹, with moisture supply of at least ~100 mm month ⁻ ¹ and ~2,000–2,500 mm yr ⁻ ¹ [7,9,13]. A caveat applies in late-century SSP5–8.5, where higher TASMAX and slightly lower RSDS increase risk of heat-related reproductive stress and spikelet abortion during sensitive seedling phases 12–24 months before harvest, implying potential declines without adaptation such as VPD-aware irrigation triggers, mulching, and heat-stress monitoring [7,63,64].

### Broader implications

Fine–scale climate and yield simulations enhance accuracy by preserving spatial gradients and short-term variability that coarse models often miss, thereby improving exposure assessments and model validation [65,66]. Our seasonal forecasting framework demonstrates clear operational benefits, offering up to eight months of lead time for farmers and cooperatives to anticipate water stress, adjust irrigation or fertilizer schedules, and plan harvest logistics [10–13]. Beyond the seasonal horizon, CMIP6-based projections provide strategic insights for long-term adaptation by pinpointing periods and locations most at risk, such as the mid-century dip in provincial yields and the central belt of Surat Thani where declines intensify. Because scenario contrasts are weak compared with timing effects, the results emphasize that adaptation should be staged over decades rather than tailored to specific emissions pathways. These projections can support regional land-use zoning, investment in irrigation infrastructure, and cultivar development programs, ensuring that Thailand’s oil palm sector remains resilient under multiple climate futures. Together, the integration of seasonal forecasts and century-scale projections creates a complementary decision-support toolkit, linking short-term operational planning with long-term strategic adaptation.

## Conclusion

This study demonstrates how fine-scale climate information can be combined with a process-guided hybrid framework to support oil palm yield forecasting and long-term assessment in a coastal and mountainous production region. A key insight is that coupling APSIM with machine learning improves predictive performance more consistently than either approach alone, because APSIM-derived physiological and soil indicators provide process-relevant structure that helps machine learning capture non-linear responses and reduce bias. This is reflected by improved error metrics and positive explanatory skill for the coupled APSIM + RF model, whereas APSIM-only and machine-learning-only approaches show limited explanatory skill when evaluated across independent locations (S7 Table).

From an application perspective, the framework links short-term and long-term decision needs. Seasonal forecasts provide actionable lead time for operational planning, while climate scenario projections support spatially explicit monitoring and adaptation planning beyond province-wide averages.

Several limitations should be noted. Evaluation is constrained by a limited number of observed yield sites and by uncertainty in precipitation and shortwave radiation downscaling, which can propagate into simulated water-balance processes. Although LOLO spatial validation reduces spatial leakage and provides a realistic estimate of transferability, the final model remains a single global model, so strong location-specific yield–climate relationships may reduce performance when extrapolating to new regions and may influence feature-importance interpretation. Management was also held constant to isolate the climate signal, so projected trajectories do not represent outcomes under active adaptation. In addition, because yields are annual time series and include multi-year carry-over effects, temporal autocorrelation may reduce the effective sample size and adds uncertainty to performance estimates, which motivates future evaluation using blocked temporal validation as longer records become available.

Future work should expand yield observations across locations and management systems, incorporate additional independent validation sources, and develop explicitly spatially varying formulations (e.g., hierarchical site effects or regional stratification) to better address non-stationarity. While we used transparent baseline models (linear regression and RF) to isolate the benefit of hybridization under a relatively small annual dataset, advanced approaches such as gradient boosting and deep learning, including transformer-based models, should be evaluated as larger and higher-frequency yield datasets become available. Probabilistic forecasting using climate and model ensembles, and scenario experiments that include realistic management adaptations and CO₂ effects, would further strengthen operational relevance.

## Supporting information

S1 Table Datasets used in this study. Downscaled climate datasets.
(DOCX)

S1 Fig Comparative analysis of RMSE across four climate variables between CHELSA, downscaled ERA5 (1980–2010), and downscaled CFSv2 datasets (2012–2016). Provincial boundaries were reproduced from Simplemaps under a CC BY 4.0 license.
(TIF)

S2 Fig Comparative analysis of RMSE across four climate variables between CHELSA and downscaled–ensembled CMIP6 datasets (1980–2010). Provincial boundaries were reproduced from Simplemaps under a CC BY 4.0 license.
(TIF)

S3 Fig Continuity diagnostic across the CHELSA to ERA5 transition for cultivated-area. The dashed vertical line marks the transition date between datasets. Values are spatial averages over cultivated pixels, with Mean ± SD annotated for each side of the transition (left segment vs right segment). TASMAX, TASMIN, and RSDS are shown at daily resolution, while PR is shown as monthly totals to reduce sensitivity to rainfall intermittency. Precipitation exhibited larger apparent junction differences than temperature and radiation because rainfall is intermittent and heavy-tailed, so monthly totals are dominated by a small number of high-intensity events and wet–dry frequency. Therefore, precipitation continuity is summarized using April monthly totals (rather than single-day comparisons) and interpreted using distribution summaries (mean, SD, median, and range), rather than expecting a near-zero difference for every year (S2 Table).
(TIF)

S2 Table Continuity check across the ERA5 to CFSv2 transition, cultivated-area mean differences between 5 and 6 April (2012–2023). Differences are computed over cultivated pixels at the concatenation window. TASMAX, TASMIN, and RSDS are evaluated at daily resolution, while PR is evaluated using April monthly totals to reduce sensitivity to rainfall intermittency and extreme events. APSIM parameterization.
(DOCX)

S3 Table APSIM oil palm Univanich’s cultivar parameterization. (DOCX)

S4 Table Fertilizer parameterization in APSIM simulations. APSIM + RF simulation.
(DOCX)

S5 Table APSIM outputs and climate variables used for GA feature selection. (DOCX)

S6 Table Features in the RF model ranked by mean absolute SHAP. (DOCX)

S7 Table Performance of APSIM and APSIM + RF across climate inputs, shown for four–site validation and site–averaged metrics. (DOCX)

S8 Table Kruskal–Wallis tests of differences in Spatially averaged ensemble yield. SPID–based downscaling computational demand. We performed SPID on an Intel(R) Core (TM) i5–13500H processor, 16.0 GB RAM, and a 64–bit operating system; it efficiently handles an area of 2 × 2 degree resolution. The initial sizes of data files were as follows: CHELSA ranged from 36 to 150 MB, ERA5 from 28 to 35 MB, CFSv2 from 0.3 to 0.4 MB, and CMIP6 from 9 to 14 MB. The downscaled ERA5 generated intermediate files of 25–50 GB with a runtime of 60–90 minutes, similar to that of downscaled CFSv2 and CMIP6. This efficiency is crucial for researchers who need to process new datasets quickly to provide timely support to practical applications.
(DOCX)
