# Supplementary material for: A machine learning–coupled APSIM model pipeline for projected oil palm yield in Surat Thani, Thailand
Source: PLoS One. 2026 Jun 10;21(6):e0349782. doi: 10.1371/journal.pone.0349782 (PMC13252752; doi:10.1371/journal.pone.0349782)
Supplement: S1 Table — Downscaled climate datasets. (DOCX) [file pone.0349782.s001.docx]

S1 Table. Datasets used in this study

| **Data type** | **Data Source** | **Parameter** | **Spatial resolution** | **Temporal resolution** |
| --- | --- | --- | --- | --- |
| Observed | TMD | Total precipitation (PR) | Station | Daily |
| Observed | TMD | Near surface maximum temperature (TASMAX) | Station | Daily |
| Observed | TMD | Near surface minimum temperature (TASMIN) | Station | Daily |
| Observed | TMD | Downward surface solar radiation (RSDS) | Station | Daily |
| Reanalysis | CHELSA | TASMAX, TASMIN, RSDS, PR | 0.03 ✕ 0.03 (3 km) | Daily |
| Reanalysis | ERA5 | TASMAX, TASMIN, RSDS, PR | 0.25 ✕ 0.25 (25 km) | Daily |
| Forecasted | CFSv2 | TASMAX, TASMIN, RSDS, PR | 1 ✕ 1 (100 km) | Daily |
| Projected | ACCESSESM1–5 | TASMAX, TASMIN, RSDS, PR | 0.25 ✕ 0.25 (25 km) | Daily |
| Projected | CNRM–CM6–1 | TASMAX, TASMIN, RSDS, PR | 0.25 ✕ 0.25 (25 km) | Daily |
| Projected | EC–Earth3–Veg | TASMAX, TASMIN, RSDS, PR | 0.25 ✕ 0.25 (25 km) | Daily |
| Projected | MPI–ESM1–2–LR | TASMAX, TASMIN, RSDS, PR | 0.25 ✕ 0.25 (27.6 km) | Daily |
| Projected | MRI–ESM2–0 | TASMAX, TASMIN, RSDS, PR | 0.25 ✕ 0.25 (27.6 km) | Daily |
| Oil palm | RSPO | Yield | Point (<1 ha) | Annually |
| Cultivated area | CROPGRIDS | Oil palm | 0.05 ✕ 0.05 (5.5 km) | – |
| Soil profile | GSDE | Sand | 0.01 ✕ 0.01 (1 km) | – |
| Soil profile | GSDE | Silt | 0.01 ✕ 0.01 (1 km) | – |
| Soil profile | GSDE | Clay | 0.01 ✕ 0.01 (1 km) | – |
| Soil profile | GSDE | Rock | 0.01 ✕ 0.01 (1 km) | – |
| Soil profile | GSDE | Bulk density (BD) | 0.01 ✕ 0.01 (1 km) | – |
| Soil profile | GSDE | Air dry | 0.01 ✕ 0.01 (1 km) | – |
| Soil profile | GSDE | Volumetric water content at –1500 kPa (LL15) | 0.01 ✕ 0.01 (1 km) | – |
| Soil profile | GSDE | Volumetric water content at –33 kPa (DUL) | 0.01 ✕ 0.01 (1 km) | – |
| Soil profile | GSDE | Volumetric water content at –10 kPa (SW) | 0.01 ✕ 0.01 (1 km) | – |
| Soil profile | GSDE | organic carbon (OC) | 0.01 ✕ 0.01 (1 km) | – |
| Soil profile | GSDE | PH | 0.01 ✕ 0.01 (1 km) | – |
