# Supplementary figures and images for: A machine learning–coupled APSIM model pipeline for projected oil palm yield in Surat Thani, Thailand
Source: PLoS One. 2026 Jun 10;21(6):e0349782. doi: 10.1371/journal.pone.0349782 (PMC13252752; doi:10.1371/journal.pone.0349782)

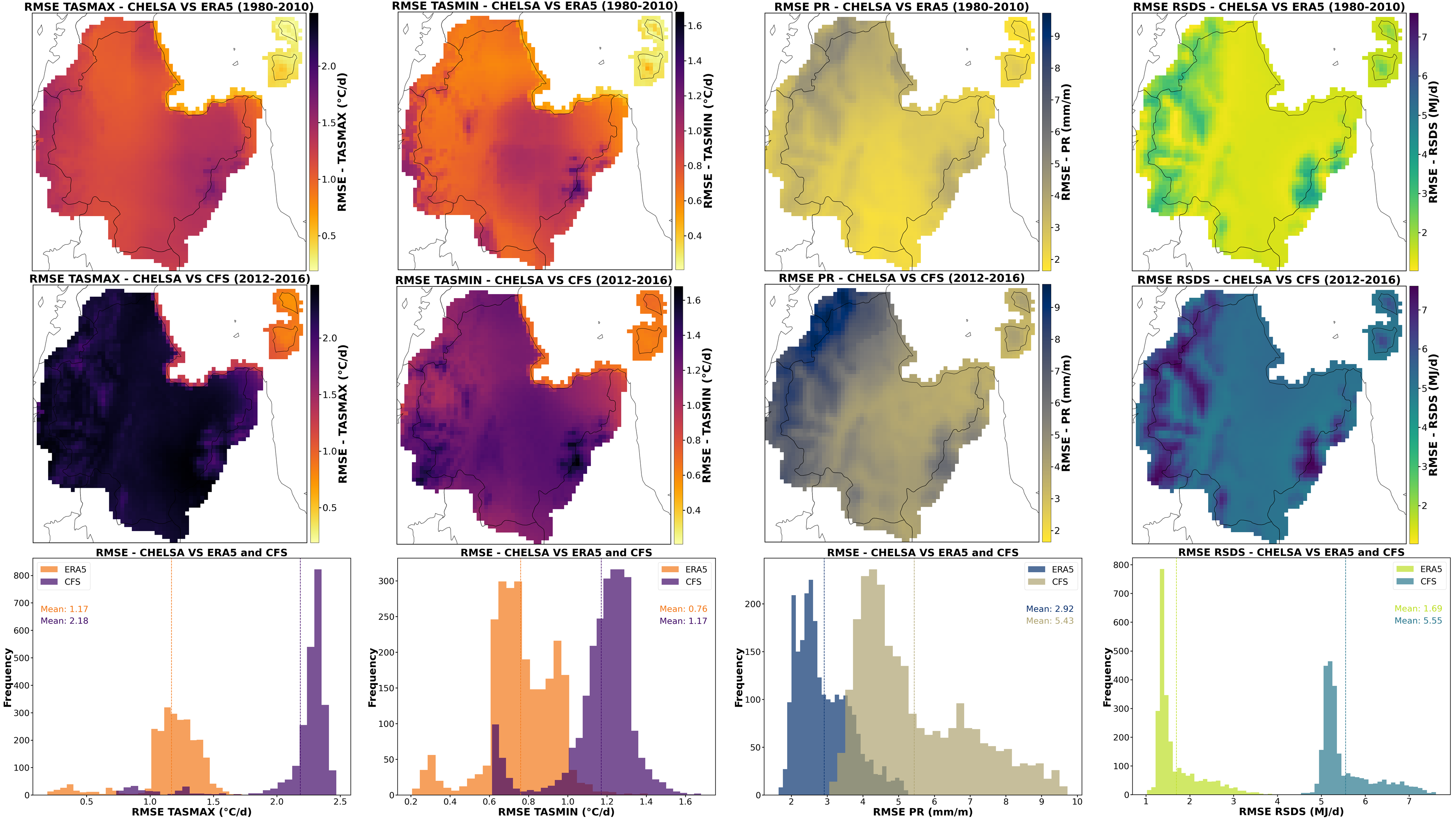

Supplement: S1 Fig — Provincial boundaries were reproduced from Simplemaps under a CC BY 4.0 license. (TIF) [file pone.0349782.s002.tif]

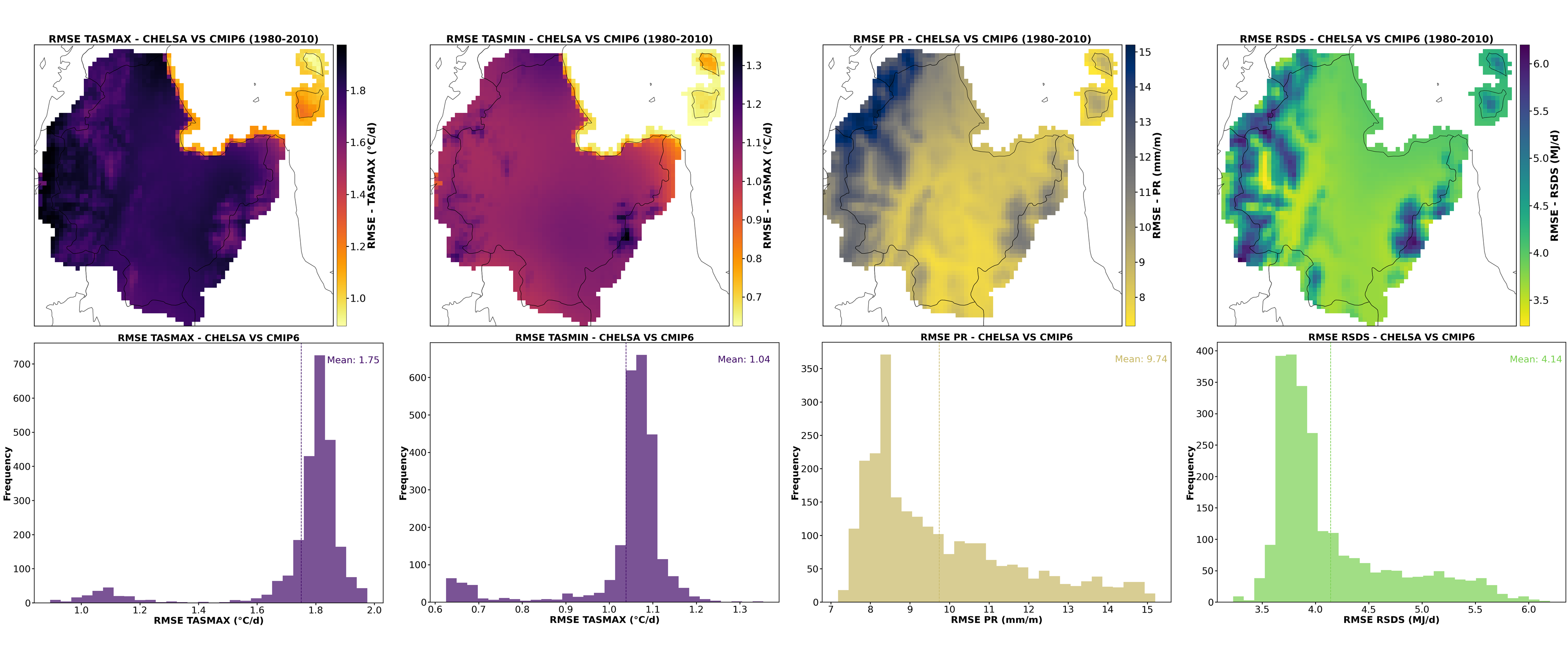

Supplement: S2 Fig — Provincial boundaries were reproduced from Simplemaps under a CC BY 4.0 license. (TIF) [file pone.0349782.s003.tif]

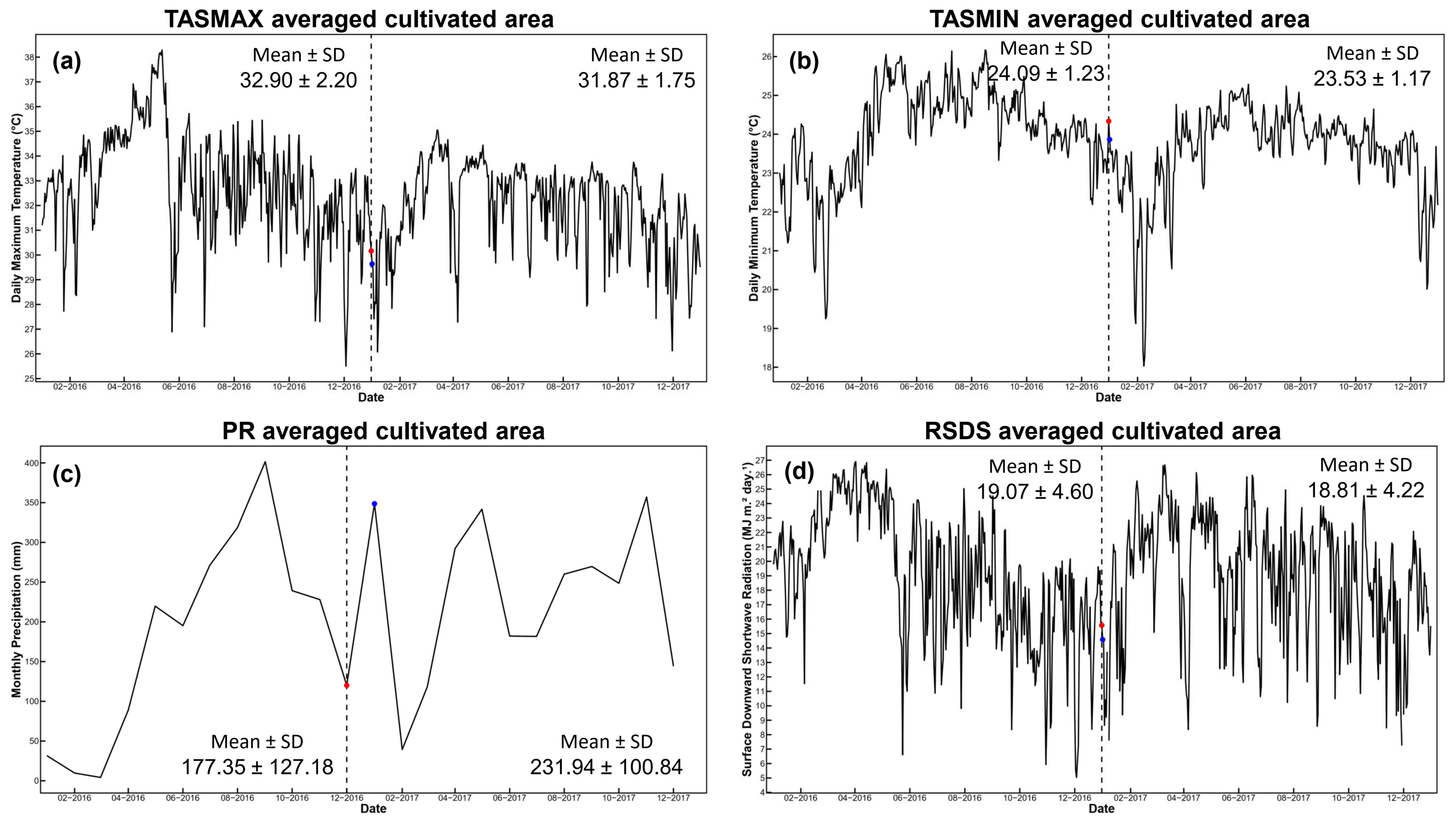

Supplement: S3 Fig — The dashed vertical line marks the transition date between datasets. Values are spatial averages over cultivated pixels, with Mean ± SD annotated for each side of the transition (left segment vs right segment). TASMAX, TASMIN, and RSDS are shown at daily resolution, while PR is shown as monthly totals to reduce sensitivity to rainfall intermittency. Precipitation exhibited larger apparent junction differences than temperature and radiation because rainfall is intermittent and heavy-tailed, so monthly totals are dominated by a small number of high-intensity events and wet–dry frequency. Therefore, precipitation continuity is summarized using April monthly totals (rather than single-day comparisons) and interpreted using distribution summaries (mean, SD, median, and range), rather than expecting a near-zero difference for every year (S2 Table). (TIF) [file pone.0349782.s004.tif]
