# Supplementary material for: A machine learning–coupled APSIM model pipeline for projected oil palm yield in Surat Thani, Thailand
Source: PLoS One. 2026 Jun 10;21(6):e0349782. doi: 10.1371/journal.pone.0349782 (PMC13252752; doi:10.1371/journal.pone.0349782)
Supplement: S2 Table — Differences are computed over cultivated pixels at the concatenation window. TASMAX, TASMIN, and RSDS are evaluated at daily resolution, while PR is evaluated using April monthly totals to reduce sensitivity to rainfall intermittency and extreme events. APSIM parameterization. (DOCX) [file pone.0349782.s005.docx]

**S2 Table. Continuity check across the ERA5 to CFSv2 transition, cultivated-area mean differences between 5 and 6 April (2012–2023). Differences are computed over cultivated pixels at the concatenation window. TASMAX, TASMIN, and RSDS are evaluated at daily resolution, while PR is evaluated using April monthly totals to reduce sensitivity to rainfall intermittency and extreme events.**

|  | **April 5 and April 6 differences** | | | |
| --- | --- | --- | --- | --- |
| **Year** | **TASMAX (°C)** | **TASMIN (°C)** | **PR (mm)** | **RSDS (MJ m⁻²)** |
| 2012 | -0.22 | -0.96 | -41.20 | 4.27 |
| 2013 | -0.82 | -0.05 | -7.29 | 9.11 |
| 2014 | 3.29 | 0.14 | -12.79 | 7.29 |
| 2015 | 1.20 | -0.87 | -13.71 | 1.13 |
| 2016 | 1.21 | -1.19 | -5.97 | 2.52 |
| 2017 | -3.65 | 0.06 | 126.79 | -14.53 |
| 2018 | 0.87 | -0.64 | 25.55 | 0.61 |
| 2019 | 0.15 | -0.75 | -71.08 | -1.37 |
| 2020 | -0.02 | -0.31 | 8.50 | 2.25 |
| 2021 | 1.18 | -0.34 | 103.64 | 2.77 |
| 2022 | 3.13 | 0.27 | 87.55 | -14.88 |
| 2023 | -0.08 | -1.39 | -85.07 | -0.22 |
| **Mean ± SD** | 0.52 ± 1.82 | -0.50 ± 0.55 | 9.57 ± 66.42 | -0.09 ± 7.44 |
| **Min, Max** | -3.65, 3.29 | -1.40, 0.27 | -85.07, 126.79 | -14.88, 9.11 |
| **Median** | 0.51 | -0.49 | -6.63 | 1.69 |
