# Supplementary material for: A machine learning–coupled APSIM model pipeline for projected oil palm yield in Surat Thani, Thailand
Source: PLoS One. 2026 Jun 10;21(6):e0349782. doi: 10.1371/journal.pone.0349782 (PMC13252752; doi:10.1371/journal.pone.0349782)
Supplement: S3 Table — (DOCX) [file pone.0349782.s006.docx]

**S3 Table. APSIM oil palm Univanich’s cultivar parameterization**

| APSIM Cultivar Parameter | Definition | At sowing (0 Year) | 5 Years | 10 Years |
| --- | --- | --- | --- | --- |
| BunchSizeMax | Maximum potential bunch weight (g) | 0 | 10,070 | 16,230 |
| FrondMaxArea | Maximum leaf area of a single frond (m²). | 0.1 | 8.14 | 9.84 |
| HarvestFrondNumber | Number of fronds retained at harvest. | 51 | 45 | 34 |
| StemToFrondFraction | Fraction of assimilates allocated to stem vs. fronds. | 0 | 0.195 | 0.2 |
| SpecificLeafAreaMax | Maximum leaf area per unit dry mass (m² g⁻¹). | – | 0.0049 | 0.0048 |
| FrondMaximumNConcentration | Maximum nitrogen concentration in fronds (%). | 1.94 | | |
