# Supplementary material for: A machine learning–coupled APSIM model pipeline for projected oil palm yield in Surat Thani, Thailand
Source: PLoS One. 2026 Jun 10;21(6):e0349782. doi: 10.1371/journal.pone.0349782 (PMC13252752; doi:10.1371/journal.pone.0349782)
Supplement: S4 Table — APSIM + RF simulation. (DOCX) [file pone.0349782.s007.docx]

**S4 Table. Fertilizer parameterization in APSIM simulations**

| Age (year) | APSIM Fertilizer | 143 trees/hectare (kg/hectare) |
| --- | --- | --- |
| At sowing | RockP | 53.625 |
| 1 | NH_4_N | 19.129396 |
| 2 | NH_4_N | 33.95392 |
| 3 | NH_4_N | 48.5056 |
| 4 | NH_4_N | 66.7381 |
| 5 | NH_4_N | 91.7648875 |
| 6 | NH_4_N | 83.422625 |
| 6+ | NH_4_N | 83.422625 |
