# Supplementary material for: A machine learning–coupled APSIM model pipeline for projected oil palm yield in Surat Thani, Thailand
Source: PLoS One. 2026 Jun 10;21(6):e0349782. doi: 10.1371/journal.pone.0349782 (PMC13252752; doi:10.1371/journal.pone.0349782)
Supplement: S5 Table — (DOCX) [file pone.0349782.s008.docx]

S5 Table. APSIM outputs and climate variables used for GA feature selection

| **APSIM Output** | | | | | **Climate Dataset** |
| --- | --- | --- | --- | --- | --- |
| **Raw** | **Raw** | **Raw** | **Raw** | **Lag adjust 0–3 year** | **Lag adjust 0–3 year** |
| AnnualAverageNO3 | AnnualUnderstoryNFixation | NO3.kgha(5) | OrganicN | Soil.Water.PAWmm(1) | TASMAX |
| AnnualBunchNPP | AnnualUnderstoryNPP | NO3.kgha(6) | SoilCtpha | Soil.Water.PAWmm(2) | TASMIN |
| AnnualET | AnnualUnderstoryNUptake | NO3.kgha(7) | SoilNkgpha | Soil.Water.PAWmm(3) | PR |
| AnnualFrondNPP | Calculations.Script.AnnualBunches | NO3.kgha(8) | TotalNitrogen | Soil.Water.PAWmm(4) | RSDS |
| AnnualPalmNUptake | Calculations.Script.AnnualBunchSize | OilPalm.Age | TotalNO3 | Soil.Water.PAWmm(5) | – |
| AnnualPalmVegetativeNPP | Calculations.Script.AnnualYield | OilPalm.BunchMass | TotalSW | Soil.Water.PAWmm(6) | – |
| AnnualPotentialEvaporation | NO3.kgha(1) | OilPalm.FrondMass | – | Soil.Water.PAWmm(7) | – |
| AnnualRootNPP | NO3.kgha(2) | OilPalm.LAI | – | Soil.Water.PAWmm(8) | – |
| AnnualStemNPP | NO3.kgha(3) | OilPalm.StemMass | – | – | – |
| AnnualTotalVegetativeNPP | NO3.kgha(4) | OrganicC | – | – | – |

Note: The numbering in parentheses (X) refers to soil layers, where layers 1–8 correspond to depths of 0–100, 100–300, 300–600, 600–900, 900–1200, 1200–1500, 1500–2000, and 2000–2500 mm, respectively.
