# Supplementary material for: A machine learning–coupled APSIM model pipeline for projected oil palm yield in Surat Thani, Thailand
Source: PLoS One. 2026 Jun 10;21(6):e0349782. doi: 10.1371/journal.pone.0349782 (PMC13252752; doi:10.1371/journal.pone.0349782)
Supplement: S6 Table — (DOCX) [file pone.0349782.s009.docx]

S6 Table. Features in the RF model ranked by mean absolute SHAP

| **Rank** | **Feature** | **Mean absolute SHAP** | **Relative (%)** |
| --- | --- | --- | --- |
| 1 | Solar radiation (MJ m⁻² yr⁻¹) (lag 1 year) | 0.55 | 12.51 |
| 2 | Maximum temperature (°C) (lag 3 year) | 0.34 | 7.82 |
| 3 | Oil palm bunch net primary production (kg/ha) | 0.32 | 7.26 |
| 4 | APSIM yield (t/ha) | 0.29 | 6.48 |
| 5 | Nitrate at 45 cm (kg/ha) | 0.28 | 6.39 |
| 6 | Organic nitrogen (kg/ha) | 0.27 | 6.23 |
| 7 | Nitrate at 135 cm (kg/ha) | 0.25 | 5.66 |
| 8 | Solar radiation (lag 2 year) | 0.25 | 5.63 |
| 9 | Average nitrate (kg/ha) | 0.23 | 5.28 |
| 10 | Total nitrogen (kg/ha) | 0.23 | 5.14 |
| 11 | PAW at 45 cm (mm yr⁻¹) (lag 2 year) | 0.22 | 5.04 |
| 12 | PAW at 175 cm (mm yr⁻¹) (lag 2 year) | 0.19 | 4.25 |
| 13 | Evapotranspiration (mm/year) | 0.18 | 4.19 |
| 14 | Soil organic C:P ratio | 0.18 | 4.12 |
| 15 | Minimum temperature (°C) | 0.15 | 3.47 |
| 16 | Maximum temperature (°C) (lag 2 year) | 0.15 | 3.38 |
| 17 | PAW at 75 cm (mm//mm/year) (lag 3 year) | 0.12 | 2.8 |
| 18 | Maximum temperature (°C) | 0.12 | 2.7 |
| 19 | Oil palm age (Year) | 0.07 | 1.66 |

Note: PAW = plant available water, C:P ratio = organic carbon and phosphorus ratio in soil.
