# Supplementary material for: A machine learning–coupled APSIM model pipeline for projected oil palm yield in Surat Thani, Thailand
Source: PLoS One. 2026 Jun 10;21(6):e0349782. doi: 10.1371/journal.pone.0349782 (PMC13252752; doi:10.1371/journal.pone.0349782)
Supplement: S7 Table — (DOCX) [file pone.0349782.s010.docx]

S7 Table. Performance of APSIM and APSIM + RF across climate inputs, shown for four–site validation and site–averaged metrics.

| **Metric type** | **Climate input** | **Yield Type** | **Mean yield (ton/hectare)** | **RMSE (ton/hectare)** | **RRMSE (%)** | **R^2^** |
| --- | --- | --- | --- | --- | --- | --- |
| Four sites | Reanalysis | Observed | 21.06 | – | – | – |
| Four sites | Reanalysis | APSIM | 30.19 | 15.51 | 73.67 | <0 |
| Four sites | Reanalysis | Delta bias corrected APSIM | 20.09 | 11.58 | 54.99 | <0 |
| Four sites | Reanalysis | Quantile mapping bias corrected APSIM | 20.67 | 11.54 | 54.82 | <0 |
| Four sites | Reanalysis | Linear regression | 22.79 | 6.57 | 31.20 | <0 |
| Four sites | Reanalysis | RF | 22.78 | 6.52 | 30.94 | <0 |
| Four sites | Reanalysis | APSIM + RF | 21.48 | 5.52 | 26.21 | 0.17 |
| Four sites | CFSv2 | APSIM + RF | 21.09 | 5.71 | 27.12 | 0.11 |
| Four sites | SSP126 | APSIM + RF | 20.83 | 5.83 | 27.69 | 0.08 |
| Four sites | SSP245 | APSIM + RF | 21.09 | 5.81 | 27.58 | 0.05 |
| Four sites | SSP585 | APSIM + RF | 21.07 | 5.69 | 27.03 | 0.06 |
| Averaged | Reanalysis | APSIM + RF | 21.48 | 2.74 | 13.01 | 0.35 |
| Averaged | CFSv2 | APSIM + RF | 21.09 | 3 | 14.23 | 0.23 |
| Averaged | SSP126 | APSIM + RF | 20.83 | 3.24 | 15.39 | 0.07 |
| Averaged | SSP245 | APSIM + RF | 21.09 | 3.32 | 15.75 | 0.13 |
| Averaged | SSP585 | APSIM + RF | 21.07 | 3.19 | 15.14 | 0.12 |
