# Supplementary material for: A machine learning–coupled APSIM model pipeline for projected oil palm yield in Surat Thani, Thailand
Source: PLoS One. 2026 Jun 10;21(6):e0349782. doi: 10.1371/journal.pone.0349782 (PMC13252752; doi:10.1371/journal.pone.0349782)
Supplement: S8 Table — SPID–based downscaling computational demand. We performed SPID on an Intel(R) Core (TM) i5–13500H processor, 16.0 GB RAM, and a 64–bit operating system; it efficiently handles an area of 2 × 2 degree resolution. The initial sizes of data files were as follows: CHELSA ranged from 36 to 150 MB, ERA5 from 28 to 35 MB, CFSv2 from 0.3 to 0.4 MB, and CMIP6 from 9 to 14 MB. The downscaled ERA5 generated intermediate files of 25–50 GB with a runtime of 60–90 minutes, similar to that of downscaled CFSv2 and CMIP6. This efficiency is crucial for researchers who need to process new datasets quickly to provide timely support to practical applications. (DOCX) [file pone.0349782.s011.docx]

S8 Table. Kruskal–Wallis tests of differences in Spatially averaged ensemble yield.

| **Test Type** | **Group** | **Chi–Squared** | **P–Value** |
| --- | --- | --- | --- |
| Scenario–wise | SSP1–2.6 | 10.16 | 0.0172 |
| Scenario–wise | SSP2–4.5 | 18.41 | 0.0004 |
| Scenario–wise | SSP5–8.5 | 11.30 | 0.0102 |
| Period–wise | 2014–2022 | 0.32 | 0.8517 |
| Period–wise | 2032–2040 | 5.98 | 0.0502 |
| Period–wise | 2062–2070 | 2.55 | 0.2789 |
| Period–wise | 2092–2100 | 1.11 | 0.5727 |
| All periods | All periods | 3.66 | 0.1607 |
